# Quasiparticle and optical properties of strained stanene and stanane

**DOI:** 10.1038/s41598-017-04210-w

**Published:** 2017-06-20

**Authors:** Pengfei Lu, Liyuan Wu, Chuanghua Yang, Dan Liang, Ruge Quhe, Pengfei Guan, Shumin Wang

**Affiliations:** 1 State Key Laboratory of Information Photonics and Optical Communications, Beijing University of Posts and Telecommunications, Beijing, 100876 China; 2 School of Physics and Telecommunication Engineering, Shaanxi University of Technology, Hanzhong, 723001 Shaanxi China; 3 State Key Laboratory of Functional Materials for Informatics, Shanghai Institute of Microsystem and Information Technology, Chinese Academy of Sciences, Shanghai, 200050 China; 4 School of Science, Beijing University of Posts and Telecommunications, Beijing, 100876 China; 5 Beijing Computational Science Research Center, Beijing, 100084 China; 6 Photonics Laboratory, Department of Microtechnology and Nanoscience, Chalmers University of Technology, 41296 Gothenburg, Sweden

## Abstract

Quasiparticle band structures and optical properties of two dimensional stanene and stanane (fully hydrogenated stanene) are studied by the GW and GW plus Bethe–Salpeter equation (GW-BSE) approaches, with inclusion of the spin-orbit coupling (SOC). The SOC effect is significant for the electronic and optical properties in both stanene and stanane, compared with their group IV-enes and IV-anes counterparts. Stanene is a semiconductor with a quasiparticle band gap of 0.10 eV. Stanane has a sizable band gap of 1.63 eV and strongly binding exciton with binding energy of 0.10 eV. Under strain, the quasiparticle band gap and optical spectrum of both stanene and stanane are tunable.

## Introduction

Two-dimensional (2D) materials have attracted a lot of attention owing to their potential applications in nanoelectronic devices and possibility to downscale the channel thickness at the atomic level, which can lead to the suppression of so-called short channel effects^1–3^. Stanene has been mentioned as a material for topological insulator (TI)^4^, which performs as new state of quantum matter with an insulating band gap in the bulk while conducting state at the edges protected by time reversal symmetry^5–9^. Zhu *et al*. reported the successful fabrication of 2D stanene by molecular beam epitaxy (MBE), which has prompted the development of related research, *e.g*., heterostructures of stanene and hydrogenated stanene (namely stanane)^10, 11^.

Although high potential of stanene in nanoelectronics is expected, the fundamental electronic and optical properties of stanene and related nanostructures are far from clear. (i) Sn, as one of the heavy elements that forms group IV-enes, needs to be considered with SOC effect. Ezawa pointed out that stanene belongs to quantum spin Hall (QSH) insulators due to its SOC effect^12^. The band gap of 0.3 eV opened by its large intrinsic spin-orbit interaction suggests the practical applications at room temperature for integrated circuits^13, 14^. (ii) The low-dimensional systems have strong many-body interactions due to the reduced screening and geometrical confinement. Previous studies on the fundamental band gaps of stanene and stanane are mainly at the standard density functional theory (DFT) level. DFT calculations indicate band gaps of 0.07 and 0.57 eV for stanene and stanane, respectively^9, 14, 15^. After applying the Heyd-Scuseria-Ernzerhof (HSE) hybrid functional, the value increases to 0.1 and 1.0 eV, respectively^16, 17^. (iii) For optical properties of stanene and stanane, no cathodoluminescence or photoluminescence measurements have yet been reported. This indicates that further studies are needed to explore the optical properties of them to expand their potential applications. Especially for stanane, its suitable value of band gap makes it a candidate for optoelectronic applications, such as photovoltaics^18^. More importantly, excitonic effects are expected to dominate the optoelectronic properties of 2D materials and corresponding devices. Excitonic effects need to be considered in calculating electronic and optical properties. However, to the best of our knowledge, a computational study at the GW(+BSE)+SOC level to accurately estimate the basic electronic and optical properties of stanene and stanane is still lacking. Meanwhile, it has been reported that the strain engineering is an efficient method to induce and tune the band gap^15–17, 19–22^. Zhang *et al*. predicted that the stanene films can be transformed into QSH insulator under the tensile strains^23^. However, only equiaxial (EQ) strain is considered in above studies. The effects of uniaxial strain along armchair (AC) and zigzag (ZZ) directions on stanene and stanane, which can also modify electronic properties and optical spectrum of 2D materials^24^, are unkown.

In this paper, we adopted the self-consistent GW0 (scGW0) method to describe the electronic structure of stanene and stanane, and the gap value for stanene and stanane increase to 0.10 and 1.63 eV, respectively. Noticeably, the GW gap value of stanane is significantly greater than its LDA gap value (0.51 eV). In the optical properties sections, at the GW+BSE+SOC level, we found a tight binding exciton locating at 1.54 eV with the binding energy of about 0.10 eV and a resonance exciton which results in a 0.61 eV redshift of the single-particle absorption peak at around 4.11 eV in stanane. The optical gap value change (from 0.53 eV at the LDA level to 1.63 eV at the GW+BSE level) redefines the applications range of stanane (from previous short-wavelength infrared to present near-infrared region), which makes it a compelling candidate for optoelectronic applications, such as photovoltaics and solar cell donor materials. By applying in-plane tensile strain, it is also possible to tune their QP band gaps and optical properties.

## Results

### Geometric structure

**Figure 1:**
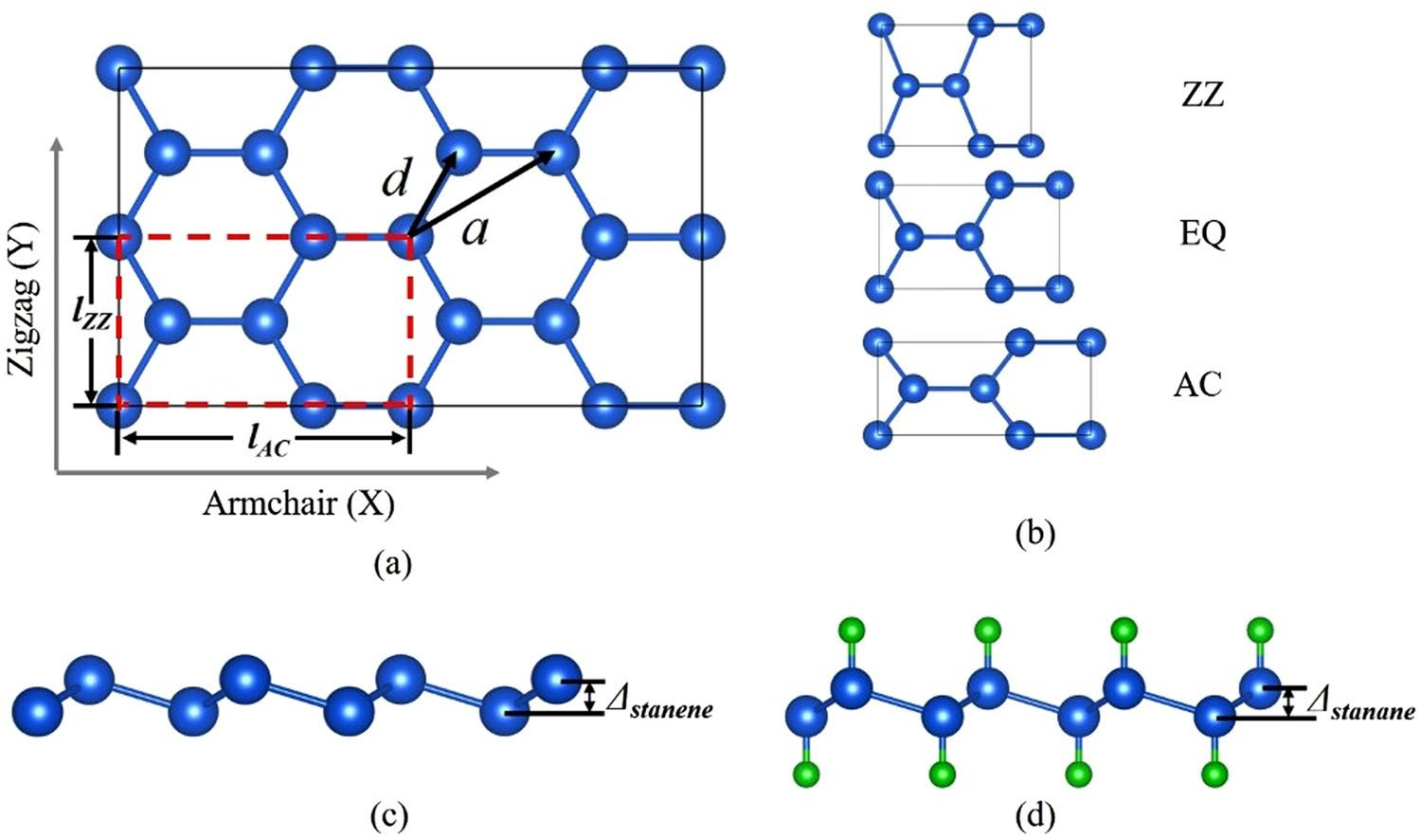
(**a**) Top view of the optimized atomic structure of stanene. The four-atom unit cell with perpendicular basis vectors is delineated by red dashed lines. (**b**) Top views of the stanene lattice under strain along ZZ, EQ and AC directions. Side views of the atomic structures of (**c**) stanene and (**d**) stanane. Blue and green balls represent Sn and H atoms, respectively.

The optimized atomic structure of stanene with the four-atom unit cell delineated by red dashed lines is shown in Fig. 1(a). The free-standing of stanene has a hexagonal honeycomb-like structure. Figure 1(b) shows the stanene atomic unit cell under three types (EQ, AC, and ZZ) of tensile strain. For the uniaxial strain, a series of incremental tensile strain are applied on the four-atom unit cell in the AC or ZZ direction and simultaneously relax the other stress components to zero.

Stanene cannot maintain the planar structure because of the tetrahedral *sp* ^3^ hybridization in Sn atoms, and a low buckled height of 0.810 Å between two Sn atom layers will be generated in Fig. 1(c). The most stable structure of stanane is the one with hydrogen atoms alternating on both sides of the Sn plane, as show in Fig. 1(d). The optimized lattice parameter of stanane is 4.567 Å, which is larger than that of stanene (4.546 Å). The stanene has a shorter Sn-Sn bond length (2.746 Å) than stanane (2.764 Å), which leads to a weaker *p*-bonding network between Sn atoms in stanane. The length of Sn-H bonds and the buckled height between two Sn atom layers in stanane are 1.722 Å and 0.830 Å, respectively.

### Electronic band structures

#### band structures of free-standing stanene and stanane

**Figure 2:**
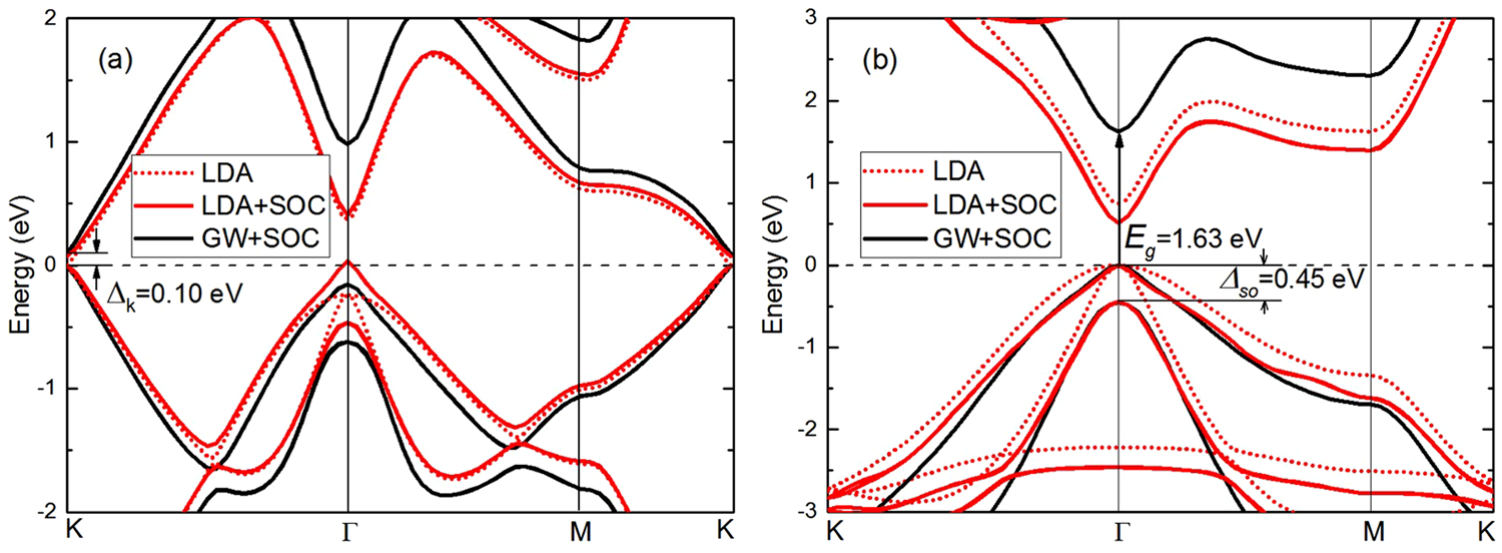
Band structure for unstrained (**a**) stanene and (**b**) stanane calculated at the LDA (red dotted), LDA+SOC (red solid), and GW+SOC (black solid) levels.

The band structure of unstrained stanene is shown in Fig. 2(a). The band gap is zero without considering SOC. Once SOC is considered, a gap of 0.07 eV is opened at the K point at the LDA+SOC level. This gap is corrected to 0.10 eV if the many body effects is considered. Previous calculation at the HSE level^16^ gives a similar gap value to our GW+SOC result (Table 1). Not only the gap at K point but also that at Г point increases after GW correction. Stanane has different band structure as shown in Fig. 2(b). After hydrogenation, the hydrogen atoms change the hybridizations of Sn atoms from *sp* ^*2*^ *-sp* ^*3*^ mixing to *sp* ^*3*^, removing the conducting *p* bands near the Fermi energy of the pure stanene. The *p* band in the pristine stanene disappears in stanane and the σ band at the Γ point becomes the top of its valence band. The free-standing stanane is a semiconductor with a direct band gap of 0.75 eV at the LDA level and the top of the valence band is degenerate. When including the SOC effect, the direct band gap changed to 0.51 eV and there emerges a spin-orbit-splitting energy (Δ_so_) of 0.45 eV at the top of valence band (Γ point). Present calculations, as well as other previous calculations, are known to underestimate the fundamental band gap. Here we apply corrections by using quasiparticle GW methods. After GW correction, the direct band gap of stanane increases to 1.63 eV, which is larger than the previous results of 1.0 eV by HSE06^17^, and the spin-orbit-splitting energy remains 0.45 eV. The previous comparative study of calculation methods has shown that the results at the GW level are more approximate to the experimental values^25^. Meantime, although hybrid functionals give reasonable value of a bulk band gap, they become unreliable in predicting the quasiparticle and optical gaps for low dimensional systems^26^. For 2D systems, the excitonic effects might be strong due to the depressed screening and reduced dimensionality. The strong excitonic effects in a material will cause the fact that the optical gap is smaller than the quasiparticle gap. By GW calculations, a quasiparticle gap with the value of 1.63 eV, which is larger than the optical gap of 1.54 eV (found below in the optical absorption spectra section), is obtained, and thus the exitonic effect is referred to be strong with a binding energy of about 0.1 eV. However, the calculated band gap (1.0 eV) of stanane at HSE level is even smaller than the optical gap (1.54 eV), which cannot account for the physical picture of excitonic effects in stanane. Therefore, to describe the quasiparticle gap and understand the excitonic effects, we need to use theory at the GW level.

**Table 1:** Comparison of the band gaps calculated at different levels of stanene and stanane (in units of eV).

Material		GGA(PBE/LDA)	HSE	GW
stanene	our work	0.07		0.10
	others	0.07^14, 15^	0.1^16^	
stanane	our work	0.51		1.63
	others	0.57^9^	1.0^17^	

#### QP band structure of strained stanene

**Figure 3:**
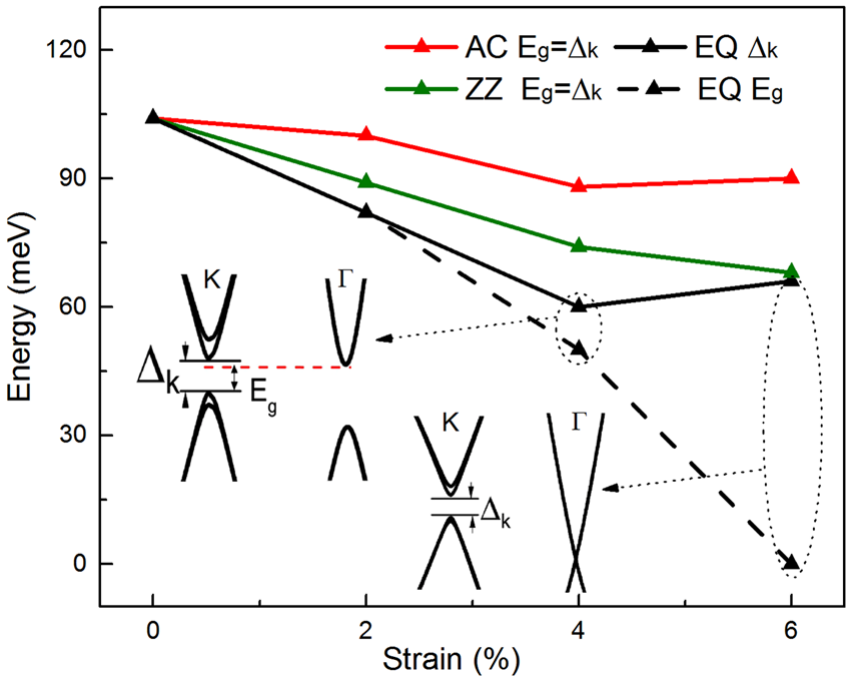
Calculated SOC-induced gap (Δ_k_) and overall band gap (*E*_g_) of stanene as fractions of biaxial strain along EQ direction, uniaxial strain along AC and ZZ directions. The two inserted figures represent the band structure of stanene under EQ 4% and 6% strain from GW calculations.

We further study the band structure of stanene under three types of strain. Like graphene and silicene, three types of strain cannot open the band gap at K point without SOC effect. Figure 3 shows calculated dependences of SOC-induced gap (Δ_k_) and overall band gap (*E*_g_) under the AC, ZZ uniaxial strain and EQ strain. The SOC-induced gap at K point decreases with all three types of strain and the extent curves of tuning are order of tens of meV. The decreasing trend of the SOC-gap in stanene under the EQ biaxial tensions has also been reported in previous LDA+SOC calculations^15^. By applying EQ tension, a semiconductor to metal transition occurs in stanene when the tension strength reaches ~6% since the gap in the Γ point closes though the SOC-induced gap in the K point remains. While for AC and ZZ uniaxial tensions, the semiconducting feature with the SOC-induced gap in the K point preserves in stanene in the checked strain range up to 6%. Therefore, the AC and ZZ uniaxial strains are effective approaches to tune the SOC-induced gap of stanene without changing its QSH state.

#### QP band structure of strained stanane

**Figure 4:**
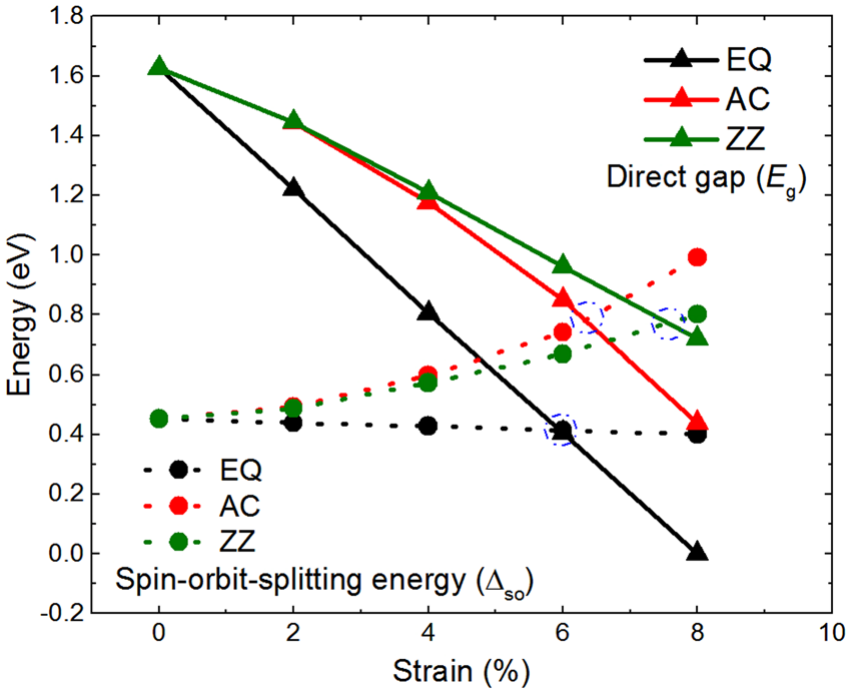
Calculated direct gap (*E*_g_) and spin-orbit-splitting energy (Δ_so_) at Γ point of stanane as fractions of biaxial strain along EQ direction, uniaxial strain along AC and ZZ directions. The solid and dash lines represent *E*_g_ and *Δ*_so_, respectively.

The dependences of the band gap (*E*_g_) and Δ_so_ under three types of strain are shown in Fig. 4. It’s clear that the direct band gap decreases with the increasing three types of strain. The decrease of direct gap shows quasi-linear feature and varies rapidly under EQ strain, while it shows nonlinear and varies slowly under uniaxial strains and slowest under ZZ direction strain. The band gap reaches almost zero when the EQ strain gets 8%, indicating that the uniaxial strain has a wide strain application on stanane.

Interestingly, the dependence of Δ_so_ exhibits different properties and a different rule between biaxial and uniaxial strain. The Δ_so_ has a weaker dependence on the biaxial strain along EQ direction, and it decreases with the increasing strain slowly. While the Δ_so_ under uniaxial strain have a much stronger dependence and show nonlinearly increase with the increasing strain. It has a much stronger dependence on AC tension and increases most quickly with increasing strain. The difference between biaxial and uniaxial strain is attributed to the change of the coupled mode of states at the top of valence-band, which results from the decrease of crystal symmetry and the inner displacement of atoms of cell after applying the AC and ZZ tensions^27–29^.

The crossover point of direct gap and Δ_so_ is marked by the blue circle as shown in Fig. 4. This is reminiscent of Auger recombination^30^. When Δ_so_ is greater than *E*_g_, Auger recombination process is forbidden and Auger loss is suppressed. The minimum tension strains to restrain Auger recombination along EQ, AC and ZZ direction are 6%, 6.3% and 7.5%, respectively.

### Excitonic effects on optical absorption spectra

#### monolayer stanene

**Figure 5:**
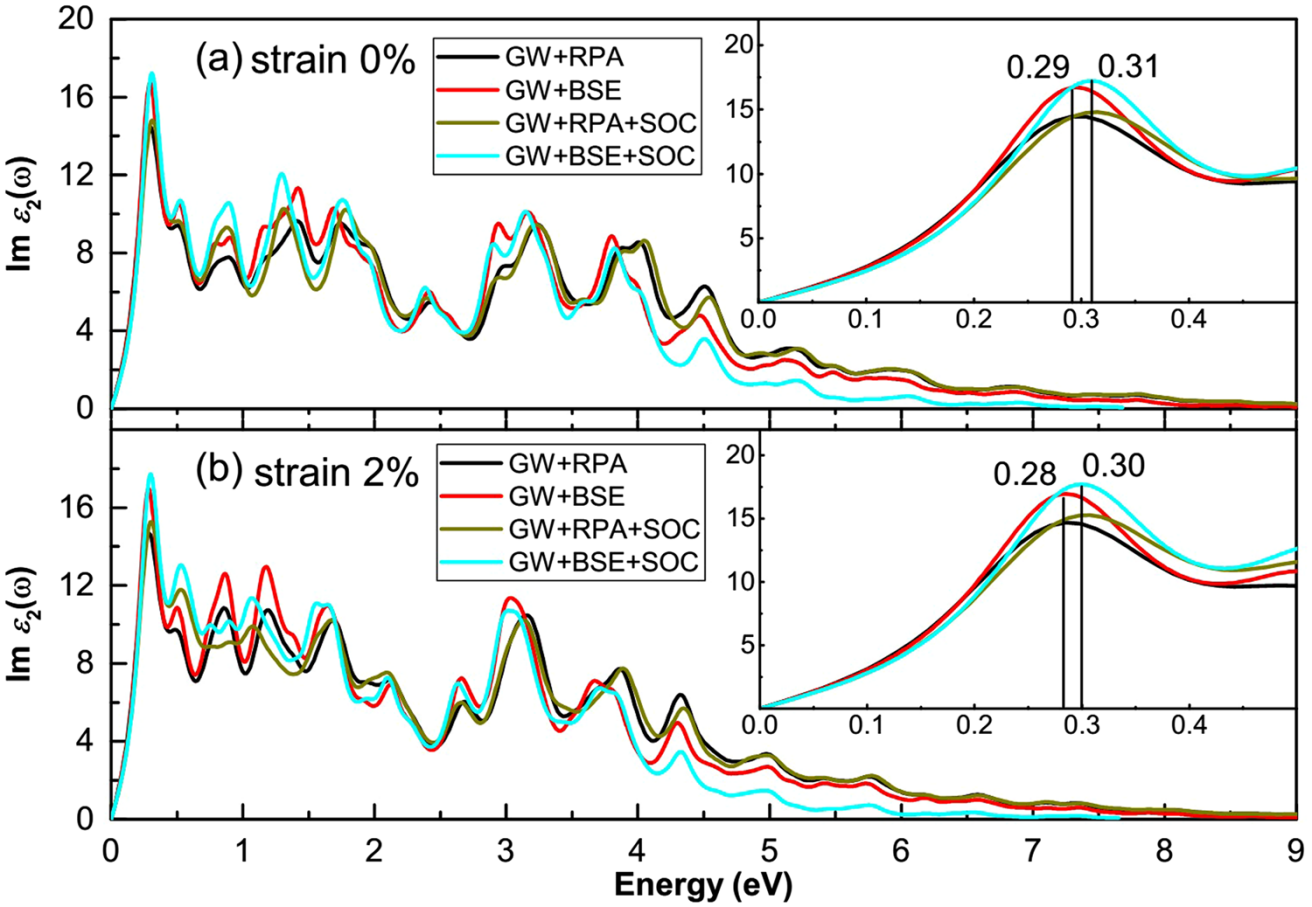
Imaginary part of dielectric functions for (**a**) 0% and (**b**) 2% EQ strained stanene with and without e–h interaction and SOC, i.e., GW+BSE, GW+RPA, GW+BSE+SOC and GW+RPA+SOC, respectively.

The absorption spectrum for stanene with and without e–h interactions are displayed in Fig. 5. Considering the depolarization effects perpendicular to the plane, we only focus on the optical absorption for light polarization parallel to the surface. Our calculations show that the optical absorption spectrum are very similar for the incident light polarized along AC and ZZ directions at both the GW+RPA and GW+BSE level. Therefore, we focus on the absorption spectrum for the incident light polarized along the AC direction in the following discussions. Meantime, considering that the band gap varies rapidly under EQ strain, we apply this particular direction strain with the value of 2% to study the strain effect on the optical properties.

In Fig. 5(a), from the line GW+RPA and GW+BSE, the optical absorptions show rapid rising from the approximately 0 eV and the first peak is the highest peak located at the 0.29 eV. This demonstrates that optical properties of stanene are analogous to the one of semimetals. The situation is due to the zero band gap of stanene at the Dirac cone. Considering the SOC effect, the absorption edge is still at approximately 0 eV and the first peak is the highest peak located at the 0.31 eV, the position and value of the first peak are weakly changed. Since the SOC effect merely induce the 0.10 eV gap, the effect of SOC on the absorption edges is not obvious. The position and value of the peaks located at higher energy have changed to some extent. This is because the electronic structure is changed after considering the SOC effect. From the iconography, we find that the BSE method with respect to the GW+RPA level merely increase the peak value of the first peak and has little effect on the shift of peak position. Unlike the semiconductors, the red-shift of first peak does not occur after considering e–h interactions. Compared with other similar 2D materials, such as silicene and germanene, stanene is likely to be a candidate of topological insulator material because of its strong SOC effect.

The imaginary part of dielectric functions of 2% EQ strained stanene is shown in Fig. 5(b). The first peaks locate at 0.28 and 0.30 eV for without and with SOC, respectively, which is slight smaller (0.01 eV) compared to the strainless condition. This demonstrates 2% strain hardly changes the rapidly rising absorption curve and the first peak.

#### Monolayer stanane

**Figure 6:**
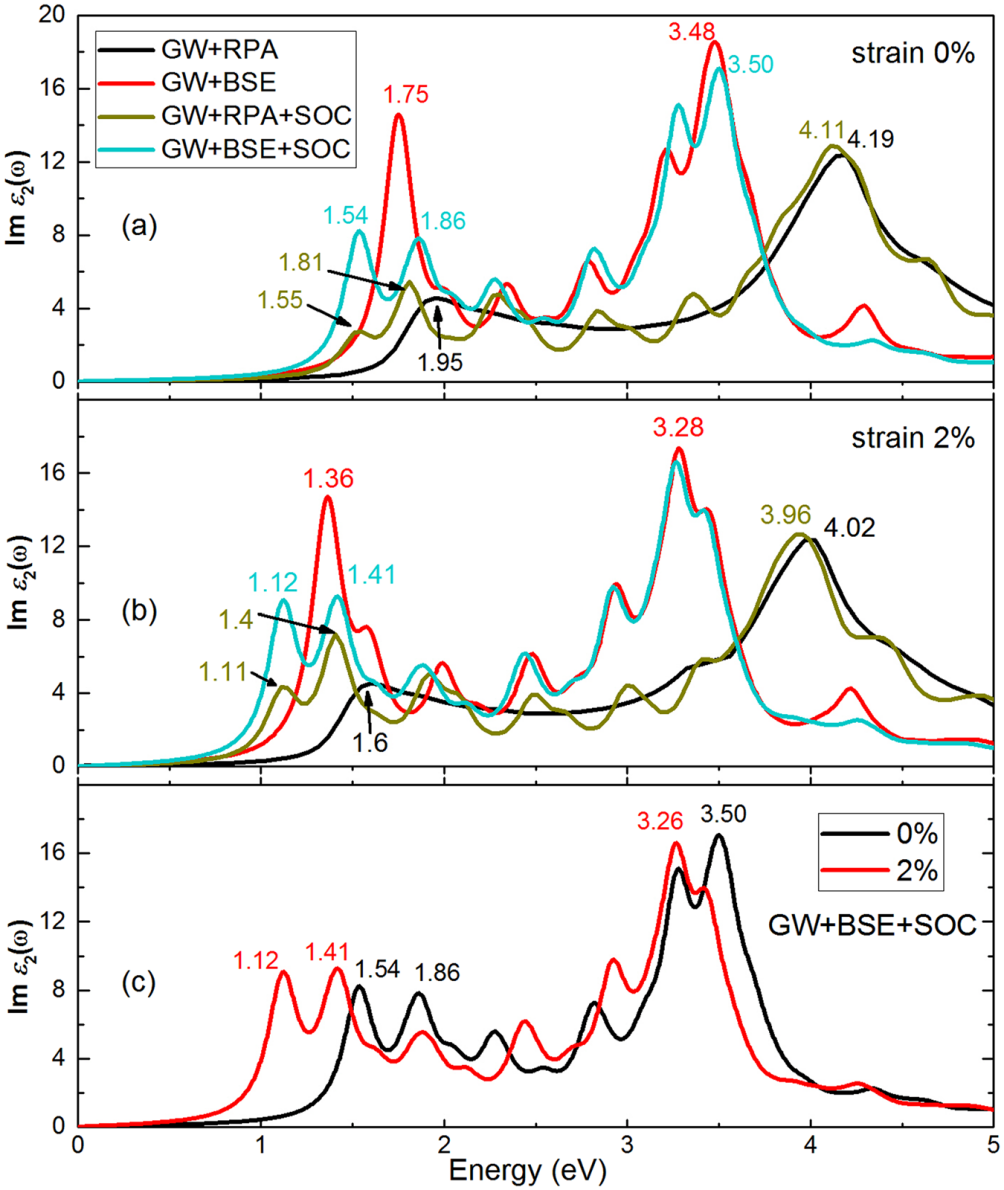
Imaginary part of dielectric functions for (**a**) 0% and (**b**) 2% EQ strained stanane without and with SOC and e–h interaction, i.e., GW+BSE, GW+RPA, GW+BSE+SOC, and GW+RPA+SOC, respectively; (**c**) Comparison between strainless and 2% EQ strained stanane.

The absorption spectrum for stanane along AC direction with and without e–h interactions are displayed in Fig. 6. Firstly, comparing GW+RPA with GW+BSE, the profile of absorption spectrum of BSE with respective to the GW+RPA level optical spectrum is reshaped. Since there is no presence of impurities, this impact should be caused by the exciton effect. The first exciton absorption peak is located at the 1.75 eV, which is smaller than 2.05 eV, which corresponds to the QP direct band gap without SOC. The binding energy, defined as the difference between exciton energy and the QP band gap, of the bound exciton is 0.30 eV. In graphane^31^, silicane^32^ and germanane^33^, the first optically excitonic state emerges at 3.80, 3.00 and 1.45 eV with binding energy of 1.6, 1.07 and 0.92 eV, respectively. Excitonic effects in stanane are weaker than that in graphane, silicane and germanane due to the different X–H binding nature (X = C, Si, Ge, Sn).

We calculated the absorption spectrum within the SOC for comparison. The absorption spectrum under the GW+RPA+SOC and GW+BSE+SOC level is shown in the Fig. 6(a). The optical spectrum is characterized by the three peaks centered on the 1.54, 1.86, 2.28, 2.82, 3.28, and 3.50 eV. The first absorption peak is observed at 1.54 eV while the absorption peak is located at 1.63 eV corresponding to the QP direct band gap in Fig. 2(b). It is clearly one tight binding exciton peaks, which is originated from two energetically degenerate excitons with an e–h binding energy of 0.09 eV. The maximum intensity of the absorption peak at the GW+BSE+SOC level is located at 3.50 eV, which is larger than the QP direct band gap 1.63 eV. It is attributed to be a resonance exciton peak and results in a 0.61 eV redshift of the single-particle absorption peak at around 4.11 eV. SOC effect makes the GW+RPA absorption band edge redshift and makes the absorption peaks split. In the lower energy range, more peaks (1.55, 1.81, 2.27, 2.82, and 3.35 eV) are split. The redshift of band edge results from the decrease of band gap when considering SOC. The splitting of absorption peak of stanane is mainly attributed to the decrease of degeneracy of band structure caused by the SOC effect. Similarly, the GW+BSE absorption spectrum changes to some extent after considering SOC. Especially, the first exciton peak (1.75 eV) is split into two exciton peaks (1.54 eV) and (1.86 eV). Meantime, we calculated the optical absorption spectrum at the LDA level with considering SOC, and found the optical gap locating at 0.53 eV. The optical gap changed to around 1.54 eV at the GW+BSE+SOC level, which covers the main energy of solar spectrum, indicates that stanene can be a promising solar cell donor materials^34^.

Within the introduction of SOC effect and BSE correction, the effects of strain on the optical absorption are calculated, as shown in Fig. 6(b). It has the same variation tendency compared with the strainless stanene. Figure 6(c) shows a comparison of imaginary part of dielectric functions between strainless stanane and 2% EQ strained stanane based on GW+BSE+SOC calculation. Compared with the strainless stanane, the basic shapes of absorption spectrum of 2% strained stanane for both GW+RPA and GW+BSE are not changed. But the whole spectrum shows an overall red-shift. This can be attributed to the quasi-linear decrease of basic band gap of stanane with the increasing EQ tensile strain. For more values of strains’ affecting on the optical properties, considering the time-consuming subjected to the computation resources, we calculated the optical properties of stanane at the LDA+RPA level and found they also show an overall red-shift.

The calculated key parameters of stanane are summarized in Table 2. The SOC effect has larger impact on the tight binding exciton than the resonance exciton. The binding energy of tight binding exciton is still approximate 0.1 eV, which indicates the 2% EQ tensile strain weakly affects the binding energy of tight binding exciton. In conclusion, both e–h interaction and SOC effect deeply affect the optical absorption of stanane. It is very indispensable to simultaneously considering the e–h interaction and SOC effect for an accurately describing the optical absorption of stanane.

**Table 2:** Intrinsic properties of monolayer stanane, including GW_0_ band gap at Γ (*E*_*g*_), first absorption peak (*E*_*f.p*_), binding energy of tight binding exciton (*E*_*tb*_), maximum intensity of the absorption peak (*E*_*m.p*_) and the redshift of *E*_*m.p*_ (*ΔE*_*m.p*_) under 0% and 2% strain with and without SOC.

Strain	SOC	GW_0_ *E*_*g*_ (eV)	BSE *E*_*f.p*_ (eV)	*E*_*tb*_ (eV)	RPA *E*_*m.p*_ (eV)	BSE *E*_*m.p*_ (eV)	*ΔE*_*m.p*_ (eV)
0%	without	2.05	1.75	0.30	4.19	3.48	0.71
	with	1.63	1.54	0.09	4.11	3.50	0.61
2%	without	1.64	1.36	0.28	4.02	3.28	0.74
	with	1.22	1.12	0.10	3.96	3.26	0.70

## Conclusion

In conclusion, we have performed first-principles calculations of the QP band structure and optical properties of stanene and stanane under strain within many-body effects and SOC effect. The GW corrected QP band gaps of free-standing stanene and stanane with SOC are 0.10 and 1.63 eV, respectively. The band gap of stanene is tunable by applying different types of strain (EQ, AC, and ZZ). Particularly, AC and ZZ uniaxial strains can tune the SOC-induced gap of stanene without changing its QSH state. The band gap of stanane decreases with all three types of tensions, and can even be closed by an 8% EQ strain. The SOC effect and e–h interactions deeply affect the optical absorption of stanane. The binding energies of tight binding exciton in stanene is 0.10 eV and the resonance exciton is found to result in a 0.61 eV redshift of the single-particle absorption peak. The suitable QP band gap and optical gap of stanane make it a compelling candidate for optoelectronic applications, such as photovoltaics and solar cell donor materials.

## Methods

Our calculations are performed by using the first-principles methods as implemented in the Vienna ab initio Simulation Package (VASP)^35^ code within the framework of DFT^36^. The exchange-correlation potential is in the form of local density approximation (LDA)^37^. The projector augmented wave (PAW)^38, 39^ method is used to describe the core electrons. Through the tests, the plane-wave basis set is defined by an energy cutoff of 400 eV for all calculations. The height of each unit cell is maintained at 25 Å to eliminate the interaction between periodic images of slabs in *z*-direction, which is sufficient to obtained a very close result to the completely isolated 2D systems. Optimal atomic positions and hexagonal structure are fully relaxed with the convergence criterion for a total energy and threshold for maximum force set as 10^−8^ eV and 10^−6^ eV/Å, respectively. For the Brillouin zone integrations, the *k*-point for the four-atom unit cell is employed with 36 × 18 × 1 Gamma-centered-grid Monkhorst-Pack *k*-mesh scheme^40^.

Over the years, the performance of GW method has been thoroughly established for bulk materials and for molecules. In comparison, serval studies on the accuracy and numerical convergence of GW calculations for 2D materials have been just reported recently^41–43^. In the following GW QP calculations, accurate scGW_0_ calculations are performed. 336 empty conduction bands are included, in which self-energy operator Σ contains almost all the electron-electron exchange and correlation effects^44–46^. Started from the scGW_0_ calculation, the QP band structure can be interpolated using the maximally localized Wannier functions (MLWFs) approach. For the Wannier band structure interpolation, *sp* ^*3*^ hybrid orbitals of Sn atom and *s* orbital of H atom are chosen for stanane’s initial projections. The *sp* ^*2*^ and *p*_*z*_ orbitals of Sn atom are used for stanene’s initial projections. Usually BSE need more *K* points than GW, a unified *K*-point mesh 36 × 36 × 1 is adopted for the GW and BSE methods, which is enough for BSE calculation. To compute the optical properties, the random-phase approximation (RPA)^47, 48^ was employed in addition to the GW approach. The attraction between quasi-electron and quasi-hole (on top of GW approximation) by solving BSE^49, 50^ is taken into account. Our BSE spectrum calculations are carried out on top of scGW_0_. The six highest valence bands and the eight lowest conduction bands are included as basis for the excitonic state. BSE was solved using the Tamm-Dancoff approximation. Simultaneously, the SOC effect is considered.
